# Precision reduction of femoral neck fractures: a novel strategy based on the femoral neck fracture morphology

**DOI:** 10.1038/s41598-024-67260-x

**Published:** 2024-07-15

**Authors:** Dongze Lin, Peisheng Chen, Chaohui Lin, Fengfei Lin

**Affiliations:** Department of Orthopaedics, Fuzhou Second General Hospital, Fuzhou Second Hospital of Xiamen University, School of Clinical Medicine of Fujian Medical University, Fujian Provincial Clinical Medical Research Center for First Aid and Rehabilitation in Orthopaedic Trauma, Fuzhou, 350007 China

**Keywords:** Medical research, Signs and symptoms

## Abstract

In femoral neck fractures the secondary damage caused by repeated multiple reductions needs to be prevented. Accordingly, the aim of this study was to achieve an anatomical reduction in the first manipulation of reduction in femoral neck fractures. We propose a new reduction strategy using the x-ray morphology of femoral neck fractures for preoperative planning. In the present study we compared this approach to conventional operation procedures for the treatment of femoral neck fractures. From 2020 to 2021, 35 patients with femoral neck fractures were operated with this reduction strategy. Those were compared with 34 patients treated without the proposed preoperative method. All patients were compared for consistency between the preoperative assessment and the intraoperative execution of the reduction approach, number of reductions, operative time, number of fluoroscopies, intraoperative bleeding, and reduction quality. The operative time of patients in the study group was significantly shorter (38.40 ± 10.26 min) than that of the control group (47.26 ± 9.09 min), and the number of reductions [1.0 (1.0, 1.0)] was significantly less than that of the control group [2.0 (1.75, 2.25)]. The number of fluoroscopies (10.27 ± 2.84) was also significantly less than that of the control group (13.53 ± 2.59) times. The KAPPA value = 0.886 shows the good agreement between the preoperative proposed protocol and the intraoperative protocol about the study group .The bleeding, quality of repositioning, Harris Hip score, MOS 12-item Short Form Survey (SF-12), and early complication rate were not statistically different between the groups (*P* > 0.05). The selection of the appropriate repositioning method based on the analysis of femoral neck fracture X-ray morphology can improve the efficiency and accuracy of preoperative planning. This reduces the secondary damage that may be caused by multiple reduction, shortens the operation time and reduces the exposure to radiation.

## Introduction

Femoral neck fractures are highly prevalent, accounting for approximately 53% of hip fractures^1–3^. There is now a consensus among surgeons that young patients with femoral neck fractures should be treated preferentially with internal fixation, the quality of reduction is one of the key factors affecting the prognosis of femoral neck fractures^4^. Even though the techniques of internal fixation of femoral neck fractures have been updated, femoral head collapse still occurs in 15–36% of cases, making the reoperation rate after fixation treatment of femoral neck fracture high^5^. Regardless of the type of fixation used and how firm the fixation is, the quality of the reduction is an independent risk factor for predicting collapse after femoral head necrosis^6^. In order to achieve anatomical repositioning of the femoral neck, many closed reduction methods have been reported to date that improve the efficiency of the reduction while reducing the incidence of postoperative collapse, re-displacement, and reoperation^7,8^. There is a fear of aggravation of displacement due to the lack of confidence in the reduction or the ability to perform an effective reduction. In situ fixation is used for Garden I femoral neck fractures. However, the morphology after femoral neck fracture varies and the direction of a displacement is three-dimensional. The clinical needs cannot be met by only one type of reduction. Up to now there are no studies comparing the best reduction method for different fracture types.

With the development of digital orthopedics, robot-assisted resetting is about to become a new option, which requires a resetting strategy based on preoperative fracture morphology. Also for femoral neck fractures, multiple intraoperative reductions should be avoided. surgeons should try to achieve anatomic repositioning on the first attempt, as repeated operations can cause secondary or even multiple injuries and destroy the residual vessels at the fracture end^9^. Developing an effective and generalized repositioning strategy is urgent as the operation is performed blindly, which can easily lead to multiple reductions.

In this retrospective study, we present a new reduction strategy based on the morphology of anterior–posterior and lateral x-ray radiographs of femoral neck fractures. We hypothesized that it would be feasible to select the appropriate repositioning method according to the fracture’s initial morphology. The intraoperative reduction should be planned preoperatively to improve the efficiency of reduction, and to reduce the repentance of reductions and operation time.

## Material and method

The study was approved by Ethics Committee of Fuzhou Second Hospital (2,021,185), conducted in accordance with the Declaration of Helsinki, and all patients signed an informed consent form. All patients signed an informed consent form. A total of 55 consecutive non-elderly patients with femoral neck fractures were included in this study from September 2020 to September 2021, 4 cases of multiple fractures and 2 cases of old fractures were excluded, and 49 patients met the inclusion criteria. The study group included: a total of 15 cases who had a mean age of 46.47 ± 12.2 years, 8 males and 7 females; 9 cases on the left side and 6 cases on the right side; Garden's classification: 6 cases of type I; 1 case of type II; 0 cases of type III; 8 cases of type IV after using the strategy of femoral neck fracture repositioning. Control group: 34 cases, 20 males and 14 females; 20 cases on the left side and 14 on the right side; age 45.85 ± 10.7 years before strategic repositioning. Garden typing: 8 cases of type I, 7 cases of type II, 7 cases of type III, 12 cases of type IV.

The inclusion criteria included: unilateral femoral neck fracture, closed fracture, age 18–65 years, follow-up time of more than six months, and fixed with FNS. Patients were excluded if they had a pathological fracture, if it was an old fracture, if there was osteoarthritis or dysplasia on the injured side of the hip joint before the injury, if other diseases affecting the patient's self-care, if they were suffering multiple fractures, or if incomplete follow-up data.

### Operative technique

All patients were fixed with a femoral neck system (FNS) for fracture fixation.

*Study group*: all patients were selected preoperatively based on the femoral neck fracture X-ray morphology according to the new repositioning strategy (including strategies: 1–4).

**Figure 1:**
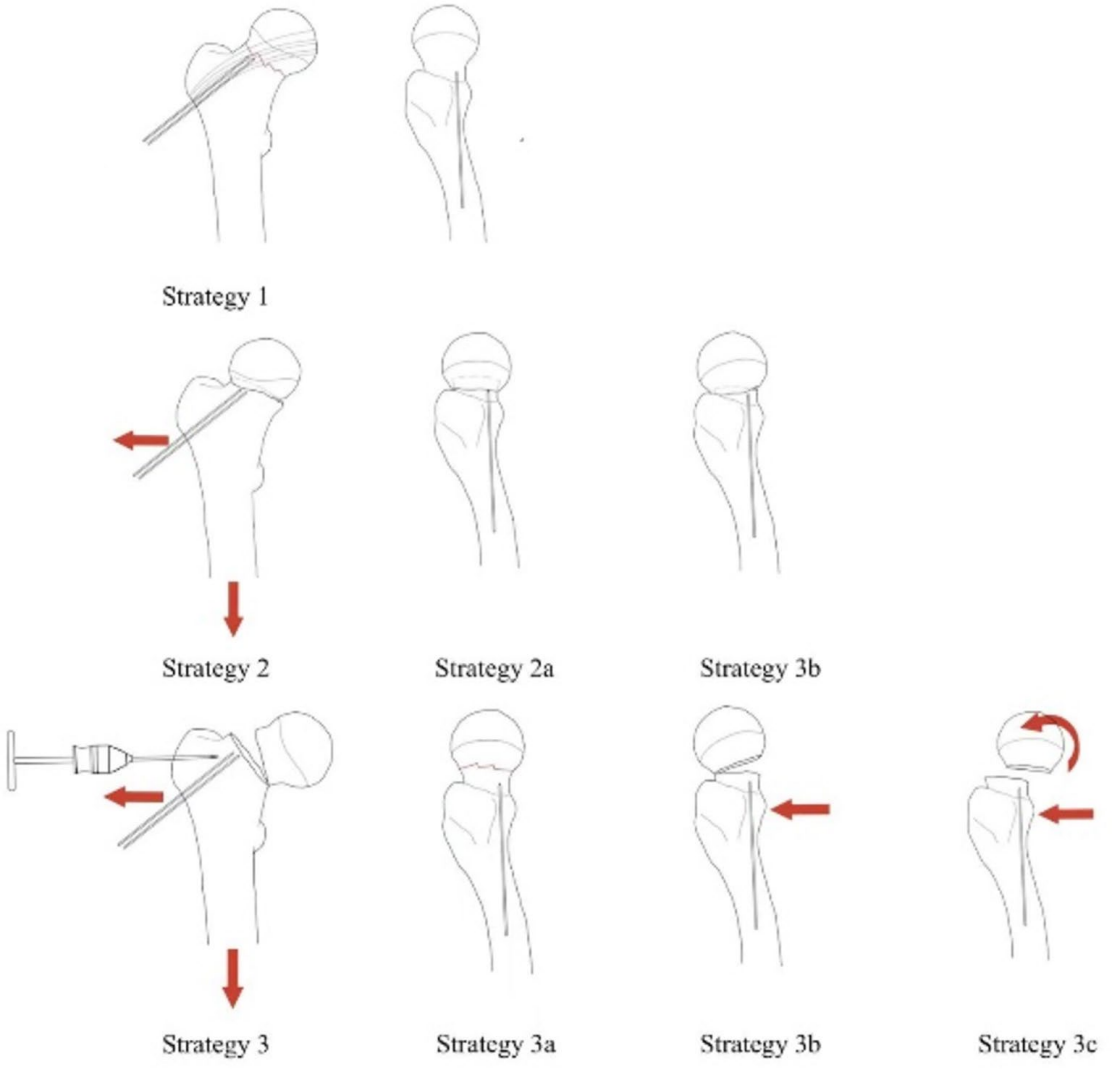
Reduction strategy I-III Strategy 1(Garden II): No displacement of bone trabeculae in anterior–posterior and lateral positions, i.e. complete fracture without displacement. Strategy 2(Garden I): Exostosis in the anterior–posterior position (angle between trabeculae and medial cortex greater than 160°) 2a: Without displacement in the lateral position. Using the bone traction screw to pull along the femoral shaft while the assistant performs joint traction at the distal end of the lower limb to release the abductor insertion of the fracture end. 2b: The lateral bone trabeculae are angled forward, and the femoral head is tilted posteriorly. Using the bone traction nail to pull along the femoral axis, in the meantime, the assistant performed joint traction on the distal end of the lower extremity, combining the forces in the three directions to release the abductor insertion of the fracture end while correcting the posterior tilt displacement of the femoral head. Strategy 3(Garden III&IV): Internal or complete displacement of the anterior and posterior trabeculae. 3a: Lateral trabeculae are not displaced. 3b: Lateral trabeculae are angled forward. 3c: Complete displacement of the lateral trabeculae. Combination of forces from four directions: bone traction, lower extremity traction, push, and Kirschner wires to control rotation to release fracture end impingement while reducing femoral head tilt and rotation.

*Reduction strategy*: intraoperative reduction is selected based on the reduction strategy (Fig. 1): the procedure is performed on a fluoroscopic orthopedic table.

*Strategy I (Garden II)*: Temporary fixation in situ with 2 Kirschner wires driven axially along the femoral neck from the lateral wall of the rotator.

*Strategy II (Garden I)*: 2a: Insert 2 Kirschner wires along the femoral neck axially from the lateral wall of the rotator, not exceeding the fracture line; insert a threaded bone traction screw 2–3 cm below the greater trochanter at the proximal femur; use the bone traction screw to pull along the femoral shaft while the assistant performs joint traction at the distal end of the lower limb to release the abductor insertion of the fracture end. 2b: Two Kirschners were driven along the femoral neck from the lateral wall, not exceeding the fracture line, and a bone traction nail was driven 2 cm-3 cm below the proximal greater trochanter of the femur, using the bone traction nail to pull along the femoral axis. Then, making a 1-2 cm incision outboard below the anterior superior iliac spine, bluntly separating to the front of the femoral neck, and using a bar for pressure to correct the posterior tilt of the femoral head (reducing with the assistance of the posterior joint capsule). While the assistant performed joint traction on the distal end of the lower extremity, combining the forces in the three directions to release the abductor insertion of the fracture end while correcting the posterior tilt displacement of the femoral head.

*Strategy III (Garden III&IV)*: 3a: The reduction method is the same as 2a. 3b: Lateral trabeculae are angled forward. The reduction method is the same as 2b. 3c: Complete displacement of the lateral trabeculae. Based on 2b, Kirschner wires avoid the weight-bearing area of the femoral head. Two 2.5 mm Kirschner wires were driven from anterior to posterior to control the rotation of the femoral head. Combination of forces from four directions: bone traction, lower extremity traction, push, and Kirschner wires to control rotation to release fracture end impingement while reducing femoral head tilt and rotation.

*Strategy IV*: Open reduction if all closed reduction fails.

All surgeries in this study were performed in anterior–posterior and lateral fluoroscopic reduction satisfactorily followed by the insertion of a preplaced Kirschner wire. Recorded the strategy of reduction and the amount of reduction.

*Control group*: Used closed reduction maneuver (by Flynn, 1974): The hip is flexed at 90° and mildly abducted. The operator pulls along the long axis of the femoral neck in the direction of the proximal thigh in one hand, fixes the knee joint in the other hand, and the assistant assists in holding the heel. The hip is straightened while maintaining traction in the long axis of the femoral neck. Straighten and internally rotate the hip joint. After satisfactory anterior–posterior and lateral fluoroscopic reduction, a preplaced Kirschner wire was inserted. For reduction requirements that could not be achieved using closed reduction maneuver, other reduction methods were selected intraoperatively based on intraoperative situation, and the reduction method and amount of reduction were recorded.

Open reduction if all closed reduction fails.

### Postoperative management

Partial weight-bearing of the affected limb is instructed from 6 to 8 weeks after surgery according to the fracture healing condition and gradually transitioned to full weight-bearing. Follow-up at the outpatient clinic at 4 weeks, 8 weeks, 12 weeks, 6 months, 12 months and 24 months postoperatively. Hip Harris score and SF-12 survival quality score are performed at 12 months and 24 months postoperatively.

Quality evaluation criteria for fracture reduction: according to the Garden index^10^ (Table 1).

**Table 1:** Garden index.

Garden index	
I	AP view: 160°, LAT view: 180°
II	AP view: 155–160°, LAT view: 180°
III	AP view: 150–155°, LAT view: > 180°
IV	AP view: < 150°, LAT view: > 180°

### Statistical analysis

SPSS 26.0 (IBM, USA) was applied for statistical analysis. Age, hospital days, follow-up time, amount of fluoroscopies, and operation time were compared using two independent samples t-test; injury to operation time was normal data with chi-square. Mann–Whitney U test was used to compare the amount of reduction and bleeding volume, Weight bearing time, Harris hip score, quality of reduction, SF-12, Mental Score (MCS) at 12 months after surgery. Gender and side of injury were compared using the χ^2^ test. The cause of injury, type of Garden, and the incidence of early complications were compared using the Fisher exact probability method. *P* < 0.05 was considered a statistically significant difference.

### Ethics approval and consent to participate

The study protocol was approved by the ethics committee of the Fuzhou Second Hospital Affiliated to Xiamen University (2,021,185). The patients gave their written informed consent for the study.

## Result

The two groups with respect of age, sex, cause of injury, side of injury, Garden's fracture classification, time from injury to surgery, days in the hospital, and follow-up time were not different (*P* > 0.05, Table 2).

**Table 2:** Demographic Characteristics of Patients in the study group vs the control group.

Characteristic	Study group (n = 15)	Control group(n = 34)	P Value
Sex			
Male	8	20	0.720
Female	7	14	
Age, Y/O	46.46 ± 12.2	45.85 ± 10.7	0.861
Causes of injury			
Low-energy	14	1	0.414
High-energy	28	6	
Time from injury to surgery, d	3.73 ± 1.86	3.41 ± 1.35	0.555
Length of stay in hospital, d	6.47 ± 2.39	7.15 ± 1.64	0.251
Follow-up interval, mth	12.07 ± 2.52	12.56 ± 2.38	0.515
Garden			
Garden I	6	8	0.114
Garden II	1	7	
Garden III	0	7	
Garden IV	8	12	

Expressed value as mean ± standard deviation.

Low-energy injury: fall from standing height; High-energy injury: traffic trauma, fall from height.

The KAPPA value = 0.886 shows the good agreement between the preoperative proposed protocol and the intraoperative protocol (Table 3).

**Table 3:** Comparison of preoperative planning and intraoperative protocol in the study group.

	Preoperative plan	Intraoperative protocol
Strategy 1	1	1
Strategy 2	6	6
Strategy 3	8	7
Strategy 4	0	1
KAPPA value	0.886	

**Figure 2:**
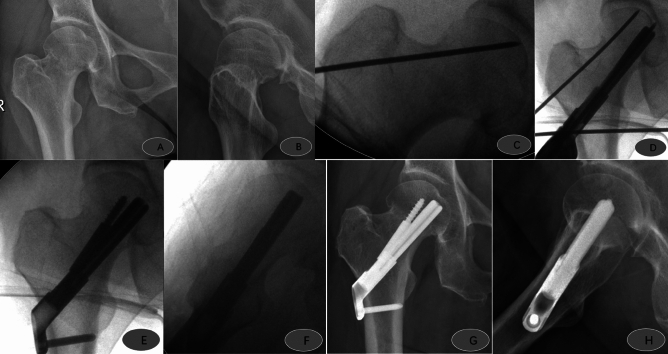
Male, 24 years old, admitted to the hospital with right hip pain and limited movement due to a fall; preoperative anteroposterior (**A**) and lateral (**B**) views showed right femoral neck fracture, Garden I, type 1; in situ fixation with FNS was placed 2 days after the injury; intraoperative operations are shown in Figures (**C**, **D**, **E** and **F**). Postoperative anteroposterior (**G**) and lateral (**H**) radiographs 3 months after surgery showed good fracture healing and satisfactory internal fixation position.

**Figure 3:**
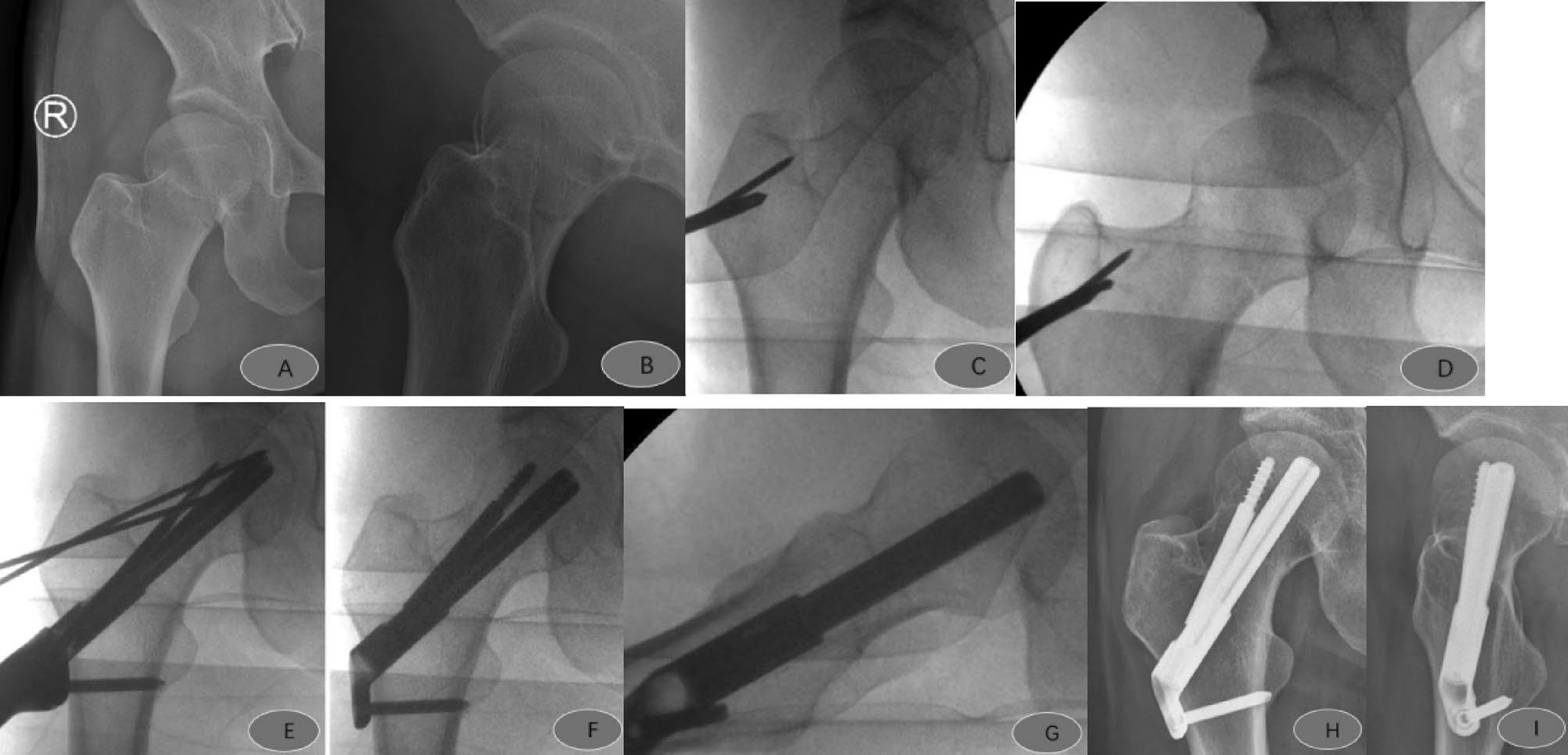
Female, 43 years old, admitted to the hospital with right hip pain and limited movement due to a fall; preoperative anteroposterior (**A**) and lateral (**B**) views show a right femoral neck fracture, Garden type I, type 2a; FNS was placed after satisfactory repositioning 5 days after the injury; intraoperative operations are shown in Figures (**C**, **D**, **E**, **F** and **G**). Postoperative anteroposterior (**H**) and lateral (**I**) radiographs 3 months after surgery show good fracture healing and satisfactory internal fixation position.

**Figure 4:**
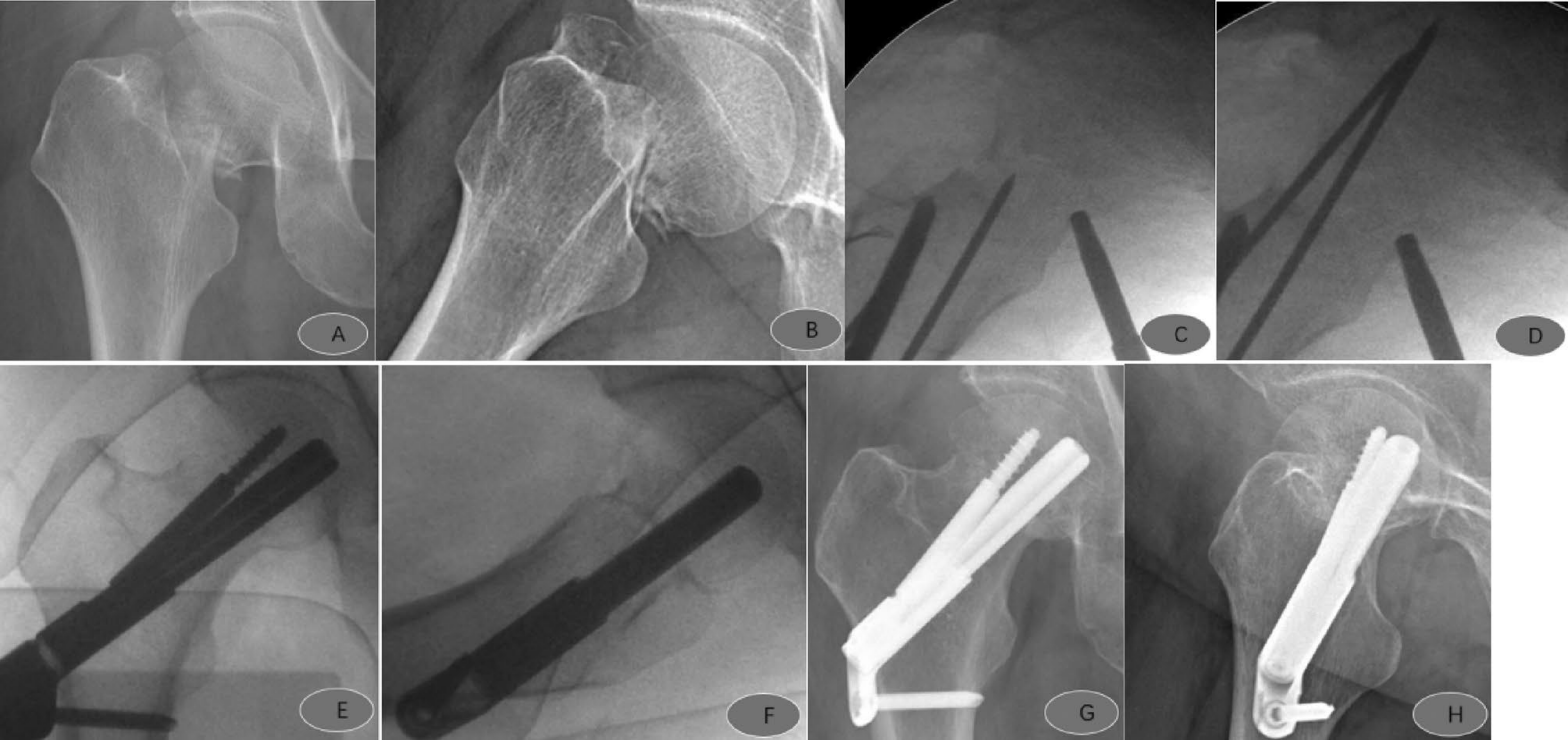
Female, 58 years old, admitted to the hospital with right hip pain and limited movement due to a fall; preoperative anteroposterior (**A**) and lateral (**B**) views showed a right femoral neck fracture, Garden type IV, type 3b; FNS was placed after satisfactory repositioning 5 days after the injury; intraoperative operations are shown in Figures (**C**, **D**, **E**, and **F**). Postoperative anteroposterior (**F**) and lateral (**H**) radiographs 3 months after surgery showed good fracture healing and satisfactory internal fixation position.

**Figure 5:**
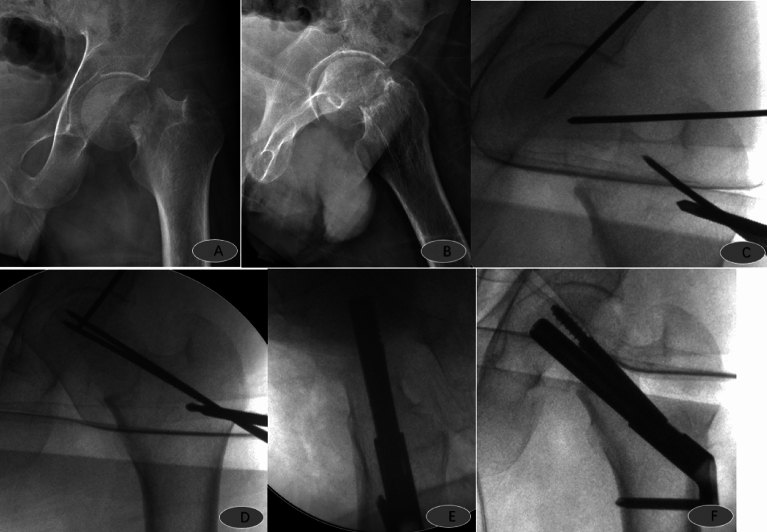
Male, 39 years old, admitted to the hospital with left hip pain and limited movement due to a fall; preoperative anteroposterior (**A**) and lateral (**B**) views show left femoral neck fracture, Garden type IV, type 3c; FNS was placed after satisfactory repositioning 1 day after the injury; intraoperative operations are shown in (**C**, **D** and **E**).

The number of intraoperative reductions and fluoroscopies of patients in the study group was significantly smaller than those in the control group, and the operating time was considerably shorter than those in the control group, with statistically significant differences (*P* < 0.05, Table 4), while there was no statistically significant difference in the comparison of the amount of bleeding, reduction quality, and early complication rate (*P* > 0.05, Table 5). In the study group, there was one case of pulmonary infection; in the control group, there were two cases of pulmonary infection, one case of lower limb deep vein thrombosis, and one case of internal fixation cut out (Table 6). Typical examples of using the reduction strategy: Figs. 2, 3, 4 and 5.

**Table 4:** Outcomes.

Outcomes	Study group (n = 15)	Control group (n = 34)	*P* value
Number of intraoperative reductions	1.0 (1.0, 1.0)	2.0 (1.75, 2.25)	< 0.001
Operative time	38.40 ± 10.26	47.26 ± 9.09	< 0.01
Amount of bleeding	50 (20,100)	28 (20, 50)	0.270
Number of fluoroscopies	10.27 ± 2.84	13.53 ± 2.59	< 0.001
Reduction quality			
Garden I	5	4	0.119
Garden II	9	27	
Garden III	1	2	
Garden IV	0	1

**Table 5:** Postoperative follow-up outcomes.

	Study group (n = 15)	Control group (n = 34)	*P* value
Harris Hip Score			
12 months after surgery	82.8 (82.4, 84.4)	82.4 (81.2, 82.8)	0.072
Weight-bearing time			
Partial weight-bearing (w)	1.0 (0, 1.0)	1.0 (0, 1.0)	0.903
Full weight-bearing (w)	6. 0 (6.0, 7.0)	6.0 (6.0, 7.0)	0.892
SF-12 survival quality score			
12 months after surgery PCS	52.32 ± 2.45	52.91 ± 3.78	0.583
12 months after surgery MCS	57.8 (56.0, 59.8)	57.8 (54.5, 59.7)	0.409

**Table 6:** Complication.

	Study group (n = 15)	Control group (n = 34)
Pulmonary infection	1	2
Lower limb deep vein thrombosis	0	1
Internal fixation cut out	0	1
Total	1	4

## Discussion

The problem of femoral head necrosis with a consecutive collapse has not been solved, so far and its incidence remains at 8.1%-37.2%^11^. Garden^12^ described the existence of a hierarchical relationship between the quality of resetting the femoral neck fractures and femoral head necrosis with collapse. The incidence of post-necrotic collapse increases as the quality of the reduction decreases. According to these findings, the garden’s alignment index was proposed, which includes 4 types. For type IV with the worst quality of reduction, the incidence of post-necrotic collapse can be up to 100%, and the concept of post-necrotic collapse of the femoral head after femoral neck fracture surgery was proposed^13^. Interestingly, the main factor causing the development of post-necrotic collapse is mechanical damage caused by concentrated stress and not for example the distribution of blood vessels. Dai^14^ noted that the average increase in stress in the head of the femur without reduction was 5.66 times higher than the stress in the head of the femur after reduction. Therefore, the anatomical reduction of femoral neck fractures to reduce the occurrence of collapse after femoral head necrosis was proposed. Poor quality of reduction after femoral neck fracture has been reported to be a risk factor for collapse after femoral head necrosis followed by an increase of reoperation rates. Accordingly, the quality of reduction can be used to predict the occurrence of post-necrotic femoral head collapse after treatment of femoral neck fractures^15^. Several studies have concluded that the quality of the reset has an impact on the development of collapse after internal fixation of femoral head necrosis in femoral neck fractures^16–19^. We believe that a collapse after femoral head necrosis is associated with local stress changes, and that displacement and shortening of the fracture end can lead to local stress changes.

Zlowodzki^20^ described the most important aspect of femoral neck fracture treatment is fracture reduction, and that improving the quality of reduction and restoring normal anatomic relationships can reduce complications. There should be only not more than two attempts of closed reductions to reduce the risk of displacement and compromised blood supply. It is also important to avoid violent reduction^21^. The current reduction techniques are mainly divided into closed reduction, limited open reduction, and open reduction^9,22^. These closed reduction techniques rely on traction and rotation of the lower extremity to achieve repositioning and are highly demanding on the surgent. Especially in Garden III and IV, which are unstable femoral neck fractures. They are often combined with cortical comminution of the severed end and rotation of the femoral head. It is difficult to achieve reduction by traction and rotation of the lower extremity. Moreover, the rotation process may lead to cortical comminution of the fracture end and even destroy the only remaining blood supply. Su^23^ reported that the percutaneous reduction is a minimally invasive reduction technique with controlled rotation of the femoral head by inserting a Kirschner wire. Reduction of the femoral head by rotation of the lower extremity has also been reported using a Kirschner wire to fix the femoral head on the acetabular side^6^. The percutaneous technique is highly advantageous for difficult femoral neck fractures. It increases the efficiency of the reduction and reduces the difficulty of the operation. However, how to improve the quality and efficiency of percutaneous reduction? We propose a reduction strategy based on the morphology of the femoral neck fracture based on the anterior–posterior and lateral X-rays of the femoral neck fracture on percutaneous reduction. The reduction strategy is resolved according to the direction of the displacement, applying traction in the opposite direction. While selecting the appropriate reduction strategy. The strategy is feasible and has been applied in clinics.

Garden classification suggests that femoral neck fractures are classified into four types, based on anterior–posterior x-ray observations of the hip joint. However, lateral x-rays were not included in the staging at that time. So that no further staging was performed for the displacement present in the lateral x-ray. In our study, we considered the quality of the lateral reduction to be equally important. Femoral neck fracture displacement is a three-dimensional directional displacement with lateral displacement and rotation. Therefore, preoperative planning and fracture reduction procedures need to consider also displacements on the lateral x-ray. Garden classification is of limited use in guiding reduction. Based on the garden classification, combined with the features of displaced femoral neck fractures on lateral x-ray.

We propose a new strategy for femoral neck fracture reduction as summarized here: We have been using in-situ fixation for non-displaced fractures on anteroposterior radiographs and lateral radiographs. For valgus impacted fractures on anteroposterior radiograph, a traction pin is used for axial traction along the femoral neck, to release the fracture end from valgus impaction. For those combined with angulation and displacement on lateral view, a tool is used to press and correct the displaced and angulated fractures. And for unstable femoral neck fractures, with rotation displacement, the rotation needs to be corrected by inserting a Kirschner wire in the non-weight-bearing area of the femoral head. When a reduction strategy is being used, all strategies can be reduced and surgically fixed in the general orthopedic surgical bed without any intraoperative traction. Avoid preoperative and post-traction differences in x-ray morphology and inconsistencies in the choice of reduction strategy because of the potential for displacement during traction.

In this study, there were statistically significant differences between the study group and the control group in terms of operative time, number of reductions, and number of fluoroscopies (*P* < 0.05), which indicates that a preoperative reduction strategy based on the morphology of femoral neck fracture can not only avoid repeated intraoperative reductions, but also simplifies the reduction process. And the reduction strategy is easy to be implemented, shortens the operative time and reduces the radiation exposure. Therefore, it is believed that the strategy can improve the reduction accuracy and efficiency. There were 5 cases (5/15, 33.3%) of Garden I reduction in the study group, and 4 cases (4/34, 11.8%) in the control group. The rate of Garden I reduction in the study group was significantly higher than that in the control group. We believe that it is the use of the reduction strategy that reduced and fixed the displaced femoral neck fracture in all three directions. However, there was no statistical difference in the quality of reduction between the two groups, which suggested that whether the reduction strategy can improve the quality of reduction needs to be included in a larger number of cases for further study. A longer follow-up of these cases is also needed to determine whether the use of a fracture morphology-based reduction strategy can improve the efficiency of reduction while reducing collapse due to pressure changes in the femoral head. Consistency statistics for the preoperative selection of the reduction method and the intraoperative protocol used had a KAPPA value of 0.886 (> 0.75). It suggests good consistency in between, indicating that the preoperative reduction strategy was helpful for intraoperative reduction.

In the clinical setting of young patients presenting with femoral neck fractures, the presence of comminution exceeding one-third of the femoral neck diameter, especially when detected through CT imaging, constitutes a considerable risk factor for the failure of internal fixation^24^. Consequently, total hip arthroplasty (THA) should be considered the preferred primary treatment modality for these patients. This treatment approach is supported by the findings of a study conducted by Melisik et al., which reported the mid-term outcomes of THA in young patients under the age of 60 with displaced intracapsular femoral neck fractures using an ultra-short anatomical cementless stem^24^. The study showed significant improvements in Harris Hip Scores and a low rate of complications, indicating that primary THA offers a robust treatment option for a rigorously selected cohort of young patients.

In conclusion, we believe that our novel strategy can improve the efficiency of reduction, reduce the damage caused by multiple reductions, shorten the operative time, and reduce the radiation exposure. The development of orthopedic surgery robots and artificial intelligence cannot be separated from repeatable and standardized processes. The morphological analysis of femoral neck fracture, combined with the reference of strategy, and the development of a standardized reduction process are believed to play a key role in the future development of digital orthopedics.

## Data Availability

The datasets used and/or analysed during the current study available from the corresponding author on reasonable request.
